# Combined pre- and post-capillary pulmonary hypertension: The clinical implications for patients with heart failure

**DOI:** 10.1371/journal.pone.0247987

**Published:** 2021-03-02

**Authors:** Tatsuro Ibe, Hiroshi Wada, Kenichi Sakakura, Yusuke Ugata, Hisataka Maki, Kei Yamamoto, Masaru Seguchi, Yousuke Taniguchi, Hiroyuki Jinnouchi, Shin-ichi Momomura, Hideo Fujita

**Affiliations:** Division of Cardiovascular Medicine, Saitama Medical Center, Jichi Medical University, Saitama, Japan; Osaka University Graduate School of Medicine, JAPAN

## Abstract

**Background:**

The prognostic implications of combined pre- and post-capillary pulmonary hypertension (Cpc-PH) in patients with pulmonary hypertension due to left heart disease (PH-LHD) remain controversial. The aim of this retrospective study was to evaluate the new PH-LHD criteria, recommended by the 6th World Symposium on Pulmonary Hypertension and to determine the prognostic value of Cpc-PH.

**Methods:**

A total of 701 patients with symptomatic heart failure who had undergone right-heart catheterization were divided into the following four groups: (i) Isolated post-capillary PH (Ipc-PH) group; mean pulmonary artery pressure (mPAP) >20 mmHg, pulmonary artery wedge pressure (PAWP) >15 mmHg, and pulmonary vascular resistance (PVR) <3 Wood units (WU) (ii) Cpc-PH group; mPAP >20 mmHg, PAWP >15 mmHg, and PVR ≥3 WU (iii) borderline-PH group; mPAP >20 mmHg and PAWP ≤15 mmHg (iv) non-PH group; mPAP ≤20 mmHg. Multivariate Cox hazard analysis was used to investigate whether Cpc-PH was associated with cardiac outcomes.

**Results:**

The study subjects were allocated into the Ipc-PH (n = 268), Cpc-PH (n = 54), borderline-PH (n = 112), or non-PH (n = 267) groups. The Cpc-PH group was associated significantly with adverse cardiac events even after adjustment for clinically relevant confounding factors for heart failure prognosis (vs. non-PH group: HR 2.98 [95% CI 1.81–4.90], *P* <0.001; vs. Ipc-PH group: HR: 1.92 [95% CI 1.19–3.08], *P* = 0.007).

**Conclusions:**

The new definitions of PH-LHD stratified patients into 4 categories. Long-term clinical outcomes were significantly different between the four categories, with Cpc-PH having the worst cardiac outcomes.

## Introduction

Pulmonary hypertension (PH) due to left heart disease (PH-LHD) caused by elevated left-sided filling pressures is the most common etiology of PH [1]. There is evidence that patients with PH-LHD have a worse clinical prognosis than patients without PH-LHD [1–3]. PH-LHD is further classified into two different subsets according to the presence of a pre-capillary component [combined pre- and post-capillary PH (Cpc-PH) or isolated post-capillary PH (Ipc-PH), respectively] [4]. Cpc-PH is considered a more serious subset than Ipc-PH [5–7]. Current ESC/ERS PH guidelines define PH as a mean pulmonary artery pressure (mPAP) ≥25 mmHg, and define the two subsets of PH-LHD according to the diastolic pressure gradient (DPG) [difference between diastolic PAP and pulmonary artery wedge pressure (PAWP)] and/or pulmonary vascular resistance (PVR) [4]. Previous studies have investigated whether or not Cpc-PH defined by current guidelines predicts clinical outcomes, although the results have varied widely in the patient groups studied [8–11]. The definition of PH using a cut-off value of mPAP ≥25 mmHg is considered empirical [12]. The categorization of two subsets of PH-LHD using DPG has also been considered too restrictive [13]. Reconsideration of these definitions is therefore warranted on the basis of recent analyses and understanding of the pathophysiology of the condition.

The 6th World Symposium on Pulmonary Hypertension suggested a major revision was needed of the new definition for PH of a mPAP >20 mmHg [12], which was based on the fact that mPAP in normal subjects was 14.0 ± 3.3 mmHg [14]. Moreover, in recent analyses [15, 16] only PVR was used as the marker to distinguish between the two subsets of PH-LHD. However, the validity of the new PH definition and categorization of two different subsets of PH-LHD by PVR has not been fully investigated. The aim of this study was to evaluate the new PH-LHD definition for risk stratification in symptomatic heart failure and to investigate the clinical outcomes of Cpc-PH defined by the new criteria.

## Methods

### Study design

We carried out a retrospective review of patients admitted to our institute. The inclusion criteria were: (1) patients with symptomatic heart failure [New York Heart Association (NYHA) functional classification ≥II and American College of Cardiology Foundation/American Heart Association (ACCF/AHA) classification Stage C or D]; (2) patients who had undergone right heart catheterization (RHC) between January 2007 and December 2016. The exclusion criteria were: (1) acute myocardial infarction, pulmonary arterial hypertension (PAH) (group 1), PH due to lung diseases and/or hypoxia (group 3), chronic thromboembolic PH (group 4), and PH with unclear and/or multifactorial mechanisms (group 5), these patients were excluded at the time of screening; (2) patients with constrictive pericarditis, congenital shunt disease, or receiving hemodialysis. The study patients were divided into four groups according to the criteria of the 6th World Symposium on Pulmonary Hypertension. The patients with symptomatic heart failure but hemodynamically categorized into pre-capillary PH were the undetermined phenotype in the new criteria. We defined this undetermined phenotype as borderline PH: (i) Ipc-PH group, mPAP >20 mmHg, PAWP >15 mmHg, and PVR <3 WU; (ii) Cpc-PH group, mPAP >20 mmHg, PAWP >15 mmHg, and PVR ≥3 WU; (iii) borderline-PH group, mPAP >20 mmHg and PAWP ≤15 mmHg; (iv) non-PH group, mPAP ≤20 mmHg. The study was approved by the institutional review board at Saitama Medical Center, Jichi Medical University (S20-145) and written informed consent was waived because of the retrospective design of the study.

### Follow-up

Clinical follow-up was performed at an office visit and by review of medical records. The follow-up period was until December 2018. The day RHC was performed was defined as the index day. The primary endpoint was the composite of cardiac death, re-admission due to heart failure, and implantation of a left ventricular assist device (LVAD). Either cardiac death, first re-admission due to heart failure, or LVAD implantation were considered as an event.

### Right heart catheterization

RHC was performed in the study subjects at the compensated stage of heart failure [8]. An external pressure transducer was zeroed at the mid-thoracic line with the patient in the supine position [17]. The average of several consecutive pressure waves over 9 seconds was recorded as the pressure measurement value during RHC. Cardiac output (CO) was measured using thermodilution with cold saline infusion.

### Definition of clinical characteristics

Left ventricular (LV) systolic function was expressed as LV ejection fraction (LVEF) measured by echocardiography and was categorized as either reduced LVEF (LVEF <50%) or preserved LVEF (LVEF ≥50%) [18]. Hypertension was defined as a past medical history of hypertension or medical treatment for hypertension before admission [19]. Diabetes mellitus was defined as a hemoglobin A1c level ≥6.5% or treatment for diabetes mellitus before admission [19]. Hyperlipidemia was defined as a low-density lipoprotein cholesterol level ≥140 mg/dL or treatment for hyperlipidemia before admission [19]. Hyperuricemia was defined as a uric acid level >7.0 mg/dL or treatment for hyperuricemia before admission [20]. Anemia was defined as a hemoglobin level <13 g/dL for men and <12 g/dL for women [21]. Renal function was evaluated by the estimated glomerular filtration rate (eGFR) using the Modification of Diet in Renal Disease formula modified for the Japanese population [22]. Impaired renal function was defined as eGFR<60 ml/min/1.73 m^2^ [19]. Estimated right ventricular systolic pressure (eRVSP) measured by echocardiography was calculated as the sum of the peak RV-right atrium (RA) gradient, while RA pressure was estimated by the diameter and respiratory change of the inferior vena cava, as reported previously [23].

### Statistical analysis

Data were expressed as mean ± standard deviation (SD) for continuous variables and frequencies and percentages for categorical variables. Analysis of normal or non-normal distributed continuous variables was performed using the Shapiro-Wilk test. Non-parametric continuous variables were analyzed using the Kruskal-Wallis test. Comparison of categorical variables in the four groups was performed using the chi-square test. Kaplan-Meier curves of the Ipc-PH, Cpc-PH, borderline-PH, and non-PH groups were constructed and the curves then compared using the log-rank test. Multivariate Cox hazard analysis was applied to investigate whether each PH group predicted cardiac death, heart failure readmission, or LVAD implantation after adjustment for confounding factors for heart failure (age [24], male sex [25], overweight [26], systolic blood pressure at admission [27], ischemic heart disease [28], anemia [29], hyperuricemia [30], impaired renal function [31], atrial fibrillation or flutter [32], reduced LVEF [33], and use of loop diuretics [34]). The statistical analyses were performed using SPSS 19/Windows statistical software (SPSS Inc, Chicago, IL, USA).

## Results

**Fig 1:**
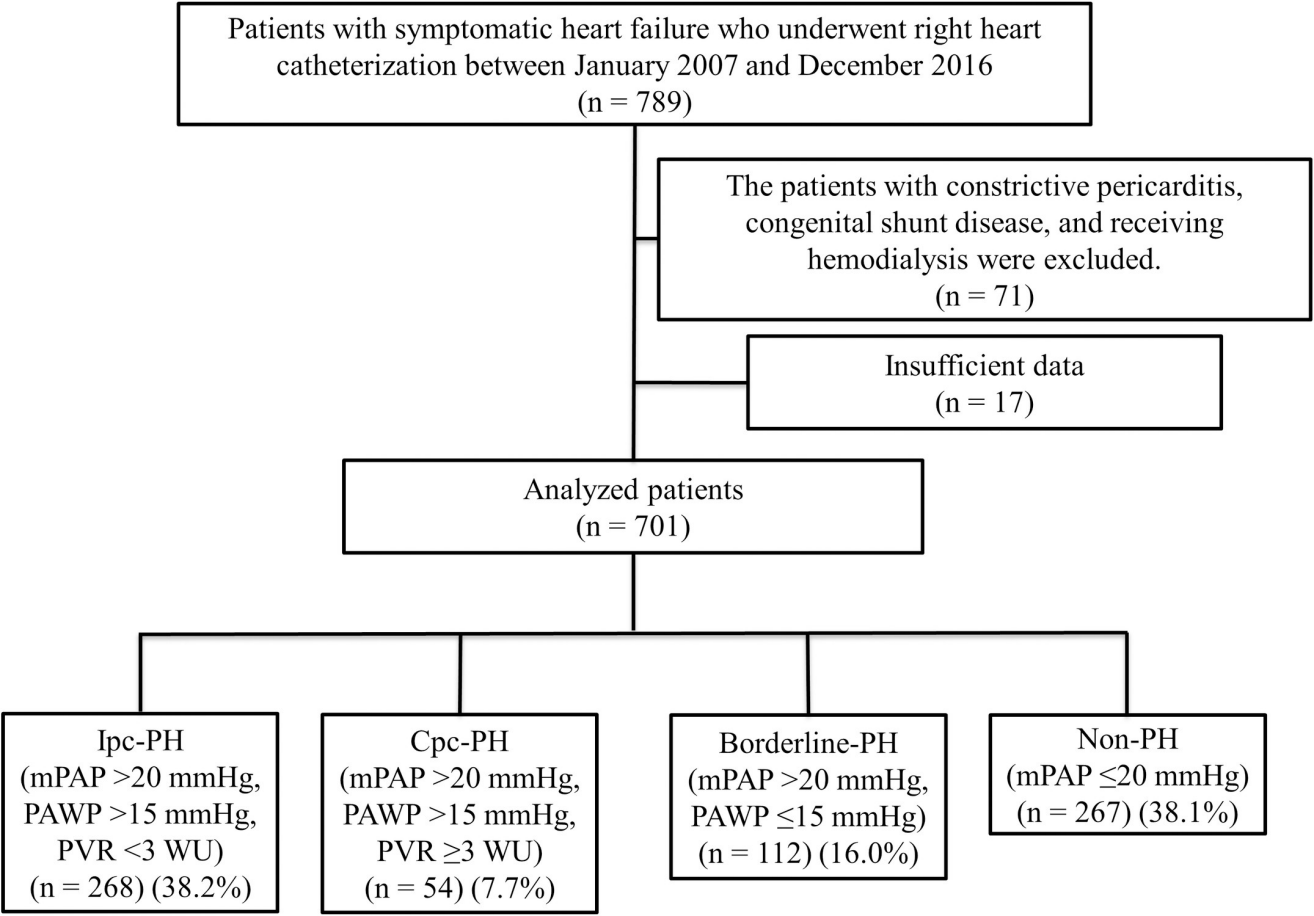
Patient enrollment. Ipc-PH, isolated post-capillary pulmonary hypertension; Cpc-PH, combined pre- and post-capillary pulmonary hypertension; PH, pulmonary hypertension; mPAP, mean pulmonary artery pressure, PAWP, pulmonary artery wedge pressure, PVR, pulmonary vascular resistance; WU, Wood units.

A total of 789 patients were admitted to our hospital for symptomatic heart failure and underwent RHC between January 2007 and December 2016. Seventy-one patients were excluded because of underlying diseases such as constrictive pericarditis, congenital shunt disease, or requirement for hemodialysis. Seventeen patients had insufficient data for RHC and were also excluded from the study. The remaining 701 patients with symptomatic heart failure were included in the analysis. Based on the values of mPAP, PAWP, and PVR the study patients were divided into the Ipc-PH (n = 268), Cpc-PH (n = 54), borderline-PH (n = 112), and non-PH (n = 267) groups. The study flow chart is shown in Fig 1.

A comparison of the clinical characteristics of the four groups is shown in Table 1. LV systolic function (reduced or preserved LVEF) was not significantly different in the four groups (*P* = 0.36). There were significant differences in the etiology of heart failure between the 4 groups, with ischemic heart disease being more common in the Cpc-PH group compared to that observed in the other groups. The eGFR level was significantly different in the 4 groups (*P* = 0.001). Over 70% of patients had received beta blockers and angiotensin converting enzyme inhibitors or angiotensin receptor blockers. The prevalence of taking loop diuretics was significantly different in the 4 groups (*P* = 0.002). The parameters of RHC are summarized in Table 2 that shows all the parameters (right atrial pressure, systolic pulmonary artery pressure, mPAP, diastolic pulmonary artery pressure, PAWP, CO, cardiac index, heart rate, PVR, DPG, and transpulmonary pressure gradient) were significantly different in the four groups (*P* <0.001).

**Table 1:** Clinical characteristics.

	Ipc-PH (n = 268)	Cpc-PH(n = 54)	Borderline-PH (n = 112)	Non-PH(n = 267)	*P* value
Age (years)	62.6 ± 14.4	63.1 ± 13.0	66.5 ± 13.6	64.9 ± 12.9	0.03
Male, n (%)	193 (72.0%)	36 (66.7%)	66 (58.9%)	174 (65.2%)	0.08
BMI (kg/m^2^)	25.3 ± 5.2	25.0 ± 4.8	24.5 ± 5.1	23.3 ± 4.5	<0.001
Heart rate on admission (beat/min)	94.3 ± 28.6	87.9 ± 22.8	90.9 ± 26.8	91.8 ± 29.8 (n = 265)	0.32
Systolic blood pressure at admission (mmHg)	125.5 ± 24.2	122.9 ± 23.2	132.5 ± 25.5	135.7 ± 34.2	0.002
Left ventricular systolic function					
Reduced LVEF, n (%)	173 (64.6%)	40 (74.1%)	67 (59.8%)	171 (64.0%)	0.36
Preserved LVEF, n (%)	95 (35.4%)	14 (25.9%)	45 (40.2%)	96 (36.0%)	
Principal etiology of heart failure					
Ischemic heart disease, n (%)	28 (10.4%)	13 (24.1%)	15 (13.4%)	30 (11.2%)	0.04
Valvular heart disease, n (%)	75 (28.0%)	12 (22.2%)	31 (27.7%)	49 (18.4%)	
Cardiomyopathy, n (%)	18 (6.7%)	4 (7.4%)	5 (4.5%)	17 (6.4%)	
Others or unknown, n (%)	147 (54.9%)	25 (46.3%)	61 (54.5%)	171 (64.0%)	
Comorbidities					
Hypertension, n (%)	138 (51.5%)	31 (57.4%)	60 (53.6%)	124 (46.4%)	0.35
Diabetes mellitus, n (%)	91 (34.0%)	23 (42.6%)	45 (40.2%)	79 (29.6%)	0.11
Hyperlipidemia, n (%)	118 (44.0%)	29 (53.7%)	53 (47.3%)	108 (40.4%)	0.27
Hyperuricemia, n (%)	164 (61.2%)	35 (64.8%)	61 (54.5%)	119 (44.6%)	0.001
COPD, n (%)	7 (2.6%)	1 (1.9%)	4 (3.6%)	6 (2.2%)	0.88
Anemia, n (%)	90 (33.6%)	13 (24.1%)	36 (32.1%)	79 (29.6%)	0.50
Impaired renal function (eGFR <60 ml/min/1.73 m^2^), n (%)	135 (50.4%)	28 (51.9%)	63 (56.3%)	114 (42.7%)	0.08
Atrial fibrillation or flutter, n (%)	122 (45.5%)	16 (29.6%)	52 (46.4%)	105 (39.3%)	0.09
Echocardiographic characteristics					
LAD (mm)	52.8 ± 8.9 (n = 263)	53.1 ± 6.4	51.6 ± 8.1 (n = 108)	49.3 ± 9.3 (n = 264)	<0.001
LVDd (mm)	60.4 ± 11.9 (n = 263)	61.7 ± 10.7	58.4 ± 10.2 (n = 108)	58.2 ± 9.9 (n = 264)	0.03
LVDs (mm)	48.2 ± 14.2 (n = 263)	50.4 ± 13.3	45.3 ± 13.0 (n = 107)	45.8 ± 12.0 (n = 264)	0.02
LVEF (%)	41.1 ± 18.1 (n = 263)	36.4 ± 19.4	44.5 ± 17.7 (n = 108)	42.6 ± 17.2 (n = 264)	0.02
eRVSP (mmHg)	40.0 ± 14.9 (n = 258)	53.1 ± 22.3 (n = 53)	40.6 ± 16.6 (n = 102)	31.0 ± 14.4 (n = 245)	<0.001
Laboratory data					
Hemoglobin (g/dl)	13.3 ± 2.1	13.9 ± 2.6	13.3 ± 2.2	13.4 ± 2.1	0.19
Na (mEq/l)	139.4 ± 3.7	138.5 ± 4.0	139.5 ± 3.8	139.5 ± 3.0	0.23
K (mEq/l)	4.3 ± 0.5	4.3 ± 0.4	4.3 ± 0.5	4.2 ± 0.5	0.94
eGFR (ml/min/1.73 m^2^)	58.4 ± 20.3	56.9 ± 17.0	58.2 ± 22.6	64.6 ± 22.1	0.001
Uric acid (mg/dl)	7.5 ± 2.4 (n = 267)	7.9 ± 2.3 (n = 53)	7.4 ± 2.4 (n = 111)	6.7 ± 1.9 (n = 266)	<0.001
BNP (pg/ml)	723.3 ± 682.8 (n = 261)	1330.1 ± 1354.9	927.9 ± 1127.2 (n = 108)	672.6 ± 675.2 (n = 261)	<0.001
Medications					
Angiotensin converting enzyme inhibitor, n (%)	146 (54.5%)	31 (57.4%)	60 (53.6%)	146 (54.7%)	0.97
Angiotensin receptor blocker, n (%)	61 (22.8%)	8 (14.8%)	29 (25.9%)	78 (29.2%)	0.10
Beta blocker, n (%)	218 (81.3%)	44 (81.5%)	84 (75.0%)	223 (83.5%)	0.29
Calcium channel blocker, n (%)	51 (19.0%)	11 (20.4%)	27 (24.1%)	54 (20.2%)	0.74
Loop diuretics, n (%)	244 (91.0%)	47 (87.0%)	94 (83.9%)	212 (79.4%)	0.002
Thiazide diuretics, n (%)	5 (1.9%)	5 (9.3%)	1 (0.9%)	11 (4.1%)	0.01
Mineralocorticoid receptor antagonist, n (%)	150 (56.0%)	36 (66.7%)	51 (45.5%)	137 (51.3%)	0.05
Digitalis, n (%)	18 (6.7%)	1 (1.9%)	9 (8.0%)	17 (6.4%)	0.49
Oral inotropic agent, n (%)	2 (0.7%)	1 (1.9%)	2 (1.8%)	1 (0.4%)	0.47
Statin, n (%)	104 (38.8%)	25 (46.3%)	54 (48.2%)	96 (36.0%)	0.11
Amiodarone, n (%)	22 (8.2%)	7 (13.0%)	9 (8.0%)	18 (6.7%)	0.49

Ipc-PH, isolated post-capillary pulmonary hypertension; Cpc-PH, combined pre- and post-capillary pulmonary hypertension; PH, pulmonary hypertension; BMI, body mass index; LVEF, left ventricular ejection fraction; COPD, chronic obstructive pulmonary disease; eGFR, estimated glomerular filtration rate; LAD, left atrium dimension; LVDd, left ventricular diastolic dimension; LVDs, left ventricular systolic dimension; eRVSP, estimated right ventricular systolic pressure; BNP, brain natriuretic peptide.

**Table 2:** Parameters of right heart catheterization.

	Ipc-PH (n = 268)	Cpc-PH(n = 54)	Borderline-PH (n = 112)	Non-PH (n = 267)	*P* value
RAP (mmHg)	10.5 ± 4.8	12.5 ± 5.4	7.2 ± 2.8 (n = 111)	5.2 ± 2.6 (n = 266)	<0.001
sPAP (mmHg)	43.2 ± 9.8	58.0 ± 14.5	35.0 ± 5.5	26.2 ± 4.7	<0.001
mPAP (mmHg)	30.3 ± 6.6	40.3 ± 8.9	23.4 ± 2.6	16.4 ± 2.8	<0.001
dPAP (mmHg)	21.8 ± 5.9	28.2 ± 7.1	15.8 ± 2.7	10.8 ± 2.6	<0.001
PAWP (mmHg)	22.6 ± 5.6	24.0 ± 5.9	12.8 ± 2.2	9.7 ± 3.0 (n = 266)	<0.001
CO (L/min)	4.9 ± 1.4	3.9 ± 1.0	5.0 ± 1.4 (n = 108)	4.8 ± 1.2 (n = 262)	<0.001
CI (L/min/m2)	2.9 ± 0.8	2.4 ± 0.7	3.1 ± 0.8 (n = 108)	2.9 ± 0.7 (n = 262)	<0.001
Heart rate (beats/min)	77.9 ± 17.5 (n = 266)	80.3 ± 13.8 (n = 53)	71.2 ± 14.2 (n = 109)	70.4 ± 14.3 (n = 256)	<0.001
PVR (Wood units)	1.6 ± 0.6	4.2 ± 1.4	2.3 ± 0.9 (n = 108)	1.5 ± 0.7 (n = 262)	<0.001
DPG (mmHg)	-0.8 ± 3.7	4.2 ± 4.7	3.0 ± 3.2	1.0 ± 2.5 (n = 266)	<0.001
TPG (mmHg	7.8 ± 3.3	16.3 ± 5.4	10.6 ± 3.2	6.7 ± 2.4 (n = 266)	<0.001

Ipc-PH, isolated post-capillary pulmonary hypertension; Cpc-PH, combined pre- and post-capillary pulmonary hypertension; PH, pulmonary hypertension; RAP, right atrial pressure; sPAP, systolic pulmonary artery pressure; mPAP, mean pulmonary artery pressure; dPAP, diastolic pulmonary artery pressure; PAWP, pulmonary artery wedge pressure; CO, cardiac output; CI, cardiac index; PVR, pulmonary vascular resistance; DPG, diastolic pressure gradient; TPG, transpulmonary pressure gradient.

**Fig 2:**
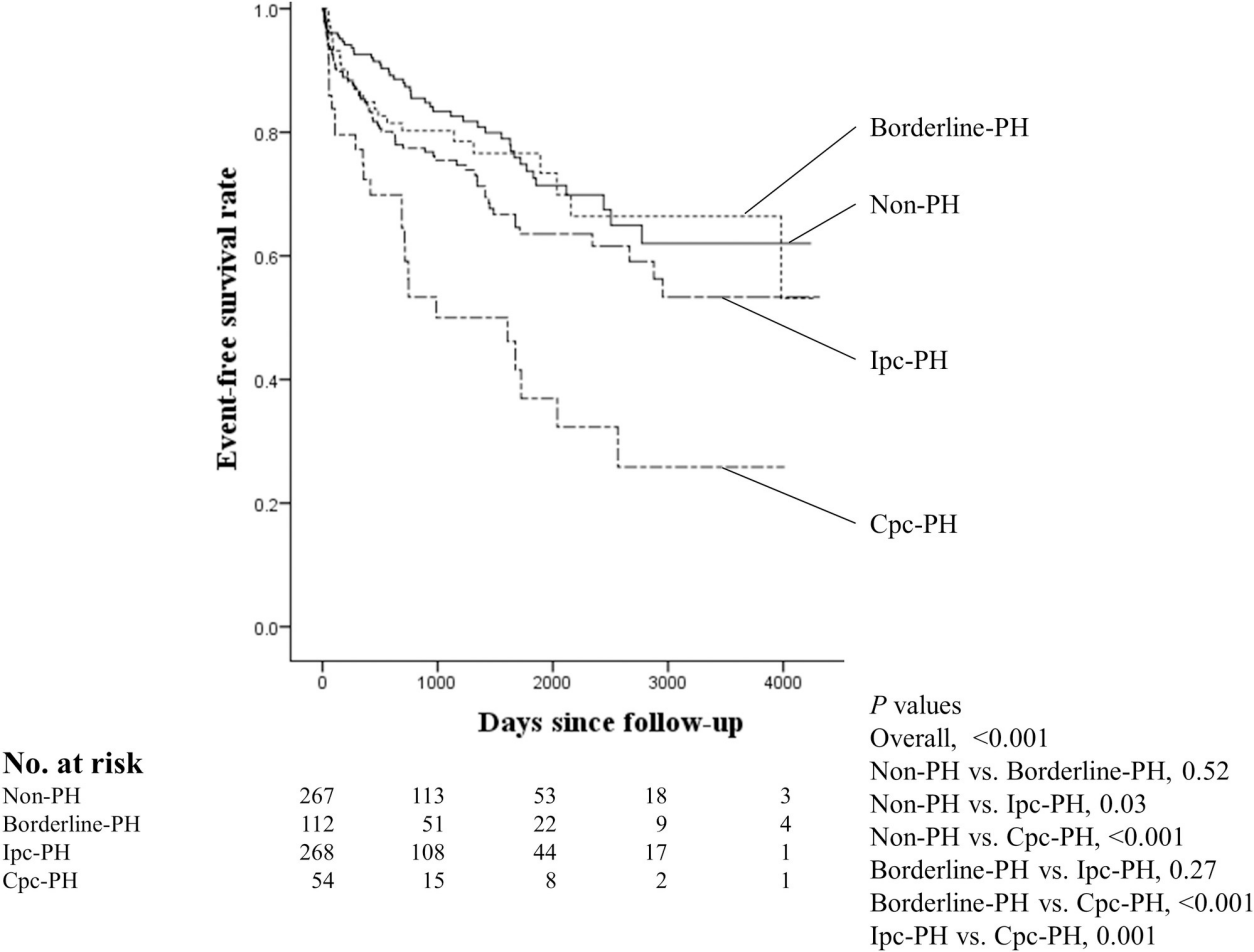
Kaplan-Meier curves for primary endpoint in the four groups. Comparison of the survival curves was performed using the log-rank test. PH, pulmonary hypertension; Ipc-PH, isolated post-capillary pulmonary hypertension; Cpc-PH, combined pre- and post-capillary pulmonary hypertension.

During a median follow-up period of 26 months, there were 166 primary endpoints (S1 Table). The Kaplan-Meier curves for the primary endpoint are shown in Fig 2. Log-rank testing revealed a significant increase in adverse events in the Ipc-PH and Cpc-PH groups compared with that in the non-PH group (*P* = 0.03 for non-PH group vs. IpC-PH group; *P* <0.001 for non-PH group vs. Cpc-PH group), while there was no significant difference between the non-PH and borderline-PH groups (*P* = 0.52). Comparison of the Ipc-PH and Cpc-PH groups showed a significant increase in the risk of adverse events in the Cpc-PH group compared with that observed in the Ipc-PH group (*P* = 0.001).

The multivariate Cox regression analysis showed a significant association between adverse cardiac events and the Ipc-PH and Cpc-PH groups compared to that occurring in the non-PH group, even after adjustment for confounding factors (Ipc-PH group, HR 1.56 [95% CI 1.06–2.29], *P* = 0.02; Cpc-PH group, HR 2.98 [95% CI 1.81–4.90], *P* <0.001) (Table 3, Model 1). In particular, the Cpc-PH group showed a significant association with cardiac events even when compared to the Ipc-PH group (HR 1.92 [95% CI 1.19–3.08], *P* = 0.007) (Table 3, Model 2).

**Table 3:** Multivariate Cox regression analysis predicting primary endpoint.

Variables	Model 1			Model 2		
	HR	95% CI	*P* value	HR	95% CI	*P* value
Classification of PH (vs. Non-PH)						
Borderline-PH	1.08	0.66–1.77	0.75			
Ipc-PH	1.56	1.06–2.29	0.02			
Cpc-PH	2.98	1.81–4.90	<0.001			
Classification of PH(vs. Ipc-PH)						
Non-PH				0.64	0.44–0.94	0.02
Borderline-PH				0.70	0.43–1.11	0.13
Cpc-PH				1.92	1.19–3.08	0.007
Age (10 year increase)	1.23	1.05–1.42	0.008	1.23	1.05–1.42	0.008
Male sex (vs. female)	0.98	0.69–1.40	0.93	0.98	0.69–1.40	0.93
Overweight (BMI ≥25 kg/m^2^)	0.99	0.70–1.39	0.94	0.99	0.70–1.39	0.94
Systolic blood pressure at admission (10 mmHg increase)	0.99	0.93–1.06	0.82	0.99	0.93–1.06	0.82
Ischemic heart disease	1.77	1.16–2.68	0.008	1.77	1.16–2.68	0.008
Anemia	1.34	0.95–1.89	0.09	1.34	0.95–1.89	0.09
Hyperuricemia	1.08	0.76–1.54	0.66	1.08	0.76–1.54	0.66
Impaired renal function (eGFR <60 ml/min/1.73 m^2^)	1.07	0.76–1.50	0.71	1.07	0.76–1.50	0.71
Atrial fibrillation or flutter	1.06	0.76–1.49	0.72	1.06	0.76–1.49	0.72
Reduced LVEF (vs. preserved LVEF)	1.21	0.84–1.74	0.32	1.21	0.84–1.74	0.32
Loop diuretics use	1.02	0.64–1.62	0.94	1.02	0.64–1.62	0.94

PH: pulmonary hypertension, Ipc-PH: isolated post-capillary pulmonary hypertension, Cpc-PH: combined pre- and post-capillary pulmonary hypertension, BMI: body mass index, eGFR: estimated glomerular filtration rate, LVEF: left ventricular ejection fraction.

## Discussion

The present study included 701 patients with symptomatic heart failure who underwent RHC and investigated whether the new definitions of PH-LHD resulted in better risk stratification of these patients. We found that the new PH-LHD definition clearly stratified patients with symptomatic heart failure and that Cpc-PH was associated significantly with adverse cardiac events even after adjustment for clinically relevant confounding factors.

### Validity for the two major changes of the new PH-LHD definition

First, we should discuss about two major issues as follows: whether a change for the mPAP cut-off value to 20 mmHg from 25 mmHg as PH was validated for PH-LHD; which changes were responsible for better prognostication, the cut-off value of mPAP 20 mmHg or using PVR instead of DPG to distinguish IpC-PH and Cpc-PH.

In addition to the fact that mPAP in normal subjects was 14.0 ± 3.3 mmHg, the change of mPAP cut-off value to 20 mmHg was mainly based on the evidence from group 1 population showing worse clinical outcomes in value of mPAP 21–24 mmHg [35, 36]. From the perspective of hemodynamic values in this study, there were few subjects with PAWP >15 mmHg and mPAP ≤20 mmHg. The proportion of PH-LHD (PAWP >15 mmHg and mPAP >20 mmHg) in total LHD (PAWP >15 mmHg) patients during the study period was 98.2%. The mean value of PAWP in the non-PH group was 9.7 ± 3.0 mmHg. Therefore, a value of 20 mmHg as the upper limit of mPAP was appropriate even in PH-LHD. On the other hand, recent study reported that short-term mortality was not statistically different between elevated and normal mPAP group in elevated PVR settings in PH-LHD [37]. We developed further survival analysis using conventional PH criteria (mPAP ≥25 mmHg) and PVR to investigate whether the change of mPAP cut-off value to 20 mmHg from 25 mmHg was meaningful for prognostication. As a result, above conventional criteria also stratified the 4 groups clearly and the Cpc-PH group showed the worst cardiac outcomes (S1 Fig and S2 Table). Thus, the change of mPAP cut-off value to 20 mmHg from 25 mmHg itself did not show meaningful change in terms of risk stratification.

As for the hemodynamic definition of Cpc-PH, there has been discussion on how to distinguish the presence of pre-capillary components in PH-LHD in recent years. The DPG had been used to distinguish between the two subsets of PH-LHD, because an elevated DPG had been shown to be linked to pulmonary vascular remodeling in PH-LHD [38]. However, there are different understanding in real world settings. Conventional PH-LHD definitions according to DPG alone did not show an association between clinical outcomes and Cpc-PH [16, 39]. By contrast, Vanderpool, et al reported that the Cpc-PH defined by elevated DPG also showed statistically significant worse clinical outcomes in a large cohort [40]. In fact, in the current study conventional PH-LHD criteria using only DPG (DPG ≥7 mmHg or not) provided the risk stratification of subjects to some extent, whereas the multivariate Cox regression analysis showed that there was no significant difference in adverse clinical outcomes observed between the Cpc-PH and Ipc-PH groups (S2 Fig and S3 Table). Several studies had revealed PVR as a predictive index for clinical outcomes in patients with heart failure [37, 39]. As for the risk stratification, conventional PH-LHD criteria using PVR alone (PVR ≥3 WU or not) could provide a clear risk stratification even after adjustment for confounding factors of heart failure in this study subjects (S1 Fig and S2 Table). Because the subjects with Cpc-PH categorized by DPG alone were too restrictive compared with that by PVR alone, misclassification of Cpc-PH would show this trend as previous study reported [16]. In fact, the multivariate Cox regression analysis showed that there was no significant difference in cardiac outcomes between the Cpc-PH and Ipc-PH groups, even in the new PH definition (mPAP >20 mmHg) and DPG (DPG ≥7 mmHg or not) (S3 Fig and S4 Table). Hence, we showed that PVR was preferable for definitions of Cpc-PH in terms of clear risk stratification.

Previously, we investigated the clinical features of PH-LHD divided by the values of DPG and transpulmonary pressure gradient (TPPG). We revealed that PH-LHD with DPG ≥7 mmHg (i.e. Cpc-PH) showed worse clinical outcomes as compared with PH-LHD with DPG <7 mmHg and TPPG ≤12 mmHg, but did not show significant difference as compared with PH-LHD with PH-LHD with DPG <7 mmHg and TPPG >12 mmHg [8]. Our previous study indicates that DPG can be a useful marker for the risk stratification of patients with PH-LHD when DPG was combined with TPPG, but DPG alone cannot be a useful marker.

Finally, we compared the outcomes of Cpc-PH between the PH-LHD definition by the “2015 ESC/ERS Guidelines for the diagnosis and treatment of pulmonary hypertension” (S4 Fig and S5 Table) and the new definition by the “6th World Symposium on Pulmonary Hypertension” (Fig 2 and Table 3). The number of patients with Cpc-PH has slightly decreased, while those with Ipc-PH and borderline-PH have increased in the new definition. The both definitions have shown worse long-term outcomes in patients with Cpc-PH compared with Ipc-PH. Notably, the new definition has clarified Cpc-PH as the worst subset of clinical outcomes even compared to other subsets. Thus, the new definition of PH-LHD more clearly stratified the patients with heart failure compared with the definition of 2015 ESC/ERS Guidelines.

### Adopt the new PH-LHD definition in real world setting

The feature of our study was that we set not all-cause death but cardiac death as one of the primary composite endpoints in order to strictly determine whether Cpc-PH was associated with heart failure-related clinical outcomes. Under the detailed study design and new definitions of PH-LHD, we found that Cpc-PH was associated significantly with worse clinical outcomes.

In previous studies, the prevalence of Cpc-PH (defined by DPG ≥7 mmHg) was approximately 12% to 13% in patients with heart failure [7, 41]. Other large cohort study showed the prevalence of Cpc-PH (defined by PVR ≥3 WU) was 36.2% within PH-LHD [40]. In our study population, Cpc-PH was 7.7% in patients with heart failure and 16.8% within PH-LHD. This prevalence of Cpc-PH was particularly low compared with above previous studies even in the new PH-LHD definition. The reason was that the severity of heart failure in our study cohort might be mild to moderate, because average mPAP value of our study was 24.7 mmHg, which was lower than average mPAP in previous studies (>30 mmHg) [7, 40]. Another difference between these previous studies and our study was that we strictly selected the study subjects. Because the severity and hemodynamics of heart failure are heterogeneous, we reviewed only patients with symptomatic heart failure. Patients with constrictive pericarditis, congenital shunt disease, and those receiving hemodialysis were excluded from the study, because PH under these conditions may not necessarily be caused by left heart disease. Strict selection of study subjects in our study showed that Cpc-PH was a less common subset of PH-LHD compared to previous studies.

As a result of the new PH-LHD definition in real world settings, patients categorized as borderline-PH represented 16.0% of the subjects in our study. Because pure pre-capillary PH such as groups 1, 3, 4, and 5 PH were not included in the study, these patients were different from pre-capillary PH and had an undetermined phenotype of PH. The borderline-PH group hemodynamically straddled the border of the non-PH and Ipc-PH group. Of course, the patients with borderline-PH might be just well compensated state, which would be the main reason that the event rates in this group showed no statistical difference when compared to the group without PH. However, the mean value of PVR and DPG in this group were 2.3 WU and 3.0 mmHg, respectively, which were higher even compared with Ipc-PH group. Although the values of PVR and DPG in borderline-PH group were close to those in Cpc-PH group, event rate of borderline-PH group straddled across non-PH and Ipc-PH groups. A recent pathological investigation in patients with heart failure with PH showed global pulmonary vascular remodeling with thickening of the media and intima in arteries and thickening of the intima in veins and small pulmonary vessels [42]. That study also showed that the severity of PH correlated most strongly with venous and small vessel remodeling [42]. When this pathological investigation applied to our study, it indicated that the relation between the degree of elevated left heart pressure and PH was not necessarily synonymous even in terms of pulmonary vein and small vessels.

One of the important issues in PH-LHD is whether Cpc-PH may benefit from specific treatment regimens. However, no study has revealed the answer to this question [43–47]. Our study showed that the new definition provided clear risk stratification for heart failure prognosis in the hemodynamically divided groups with symptomatic heart failure. Using this clear risk stratification it is possible that each subset could be different treatments targets. However, the definitive answer to this issue also requires greater understanding of the pathophysiology of PH-LHD.

### Study limitations

The study was a retrospective design in a single tertiary center, that may have resulted in higher selection of patients who were hospitalized in our center. It is also possible that incomplete follow-up may have occurred because clinical follow-up was performed at office visits and by review of medical records. Finally, we should mention that we could not clarify whether or not Cpc-PH defined by the new criteria had a pathological pre-capillary component in PH-LHD. Further prospective studies and pathological evaluations are therefore warranted.

## Conclusions

The definitions for PH-LHD of the 6th World Symposium on Pulmonary Hypertension recommendations provide clear risk stratification in symptomatic heart failure. Notably, Cpc-PH defined by the new criteria is associated significantly with worse cardiac outcomes.

## Supporting information

S1 Table Endpoints. (DOCX)

S2 Table Multivariate Cox regression analysis to predict primary endpoint using conventional PH criteria and PVR. (DOCX)

S3 Table Multivariate Cox regression analysis to predict primary endpoint using conventional PH criteria and DPG. (DOCX)

S4 Table Multivariate Cox regression analysis to predict primary endpoint using the new PH criteria and DPG. (DOCX)

S5 Table Multivariate Cox regression analysis to predict primary endpoint using the current PH-LHD definition by the 2015 ESC/ERS guidelines for the diagnosis and treatment of pulmonary hypertension. (DOCX)

S1 Fig Kaplan-Meier curves of the 4 groups stratified according to conventional PH criteria and PVR. Comparison of the survival curves was performed using the log-rank test. PH, pulmonary hypertension; PVR, pulmonary vascular resistance; Ipc-PH, isolated post-capillary pulmonary hypertension; Cpc-PH, combined pre- and post-capillary pulmonary hypertension.
(TIF)

S2 Fig Kaplan-Meier curves of the 4 groups stratified according to conventional PH criteria and DPG. Comparison of the survival curves was performed using the log-rank test. PH, pulmonary hypertension; DPG, diastolic pressure gradient; Ipc-PH, isolated post-capillary pulmonary hypertension; Cpc-PH, combined pre- and post-capillary pulmonary hypertension.
(TIF)

S3 Fig Kaplan-Meier curves of the 4 groups stratified according to the new PH criteria and DPG. Comparison of the survival curves was performed using the log-rank test. PH, pulmonary hypertension; DPG, diastolic pressure gradient; Ipc-PH, isolated post-capillary pulmonary hypertension; Cpc-PH, combined pre- and post-capillary pulmonary hypertension.
(TIF)

S4 Fig Kaplan-Meier curves of the 4 groups stratified according to the current PH-LHD definition by the 2015 ESC/ERS guidelines for the diagnosis and treatment of pulmonary hypertension. Comparison of the survival curves was performed using the log-rank test. PH-LHD, pulmonary hypertension due to left heart disease; PH, pulmonary hypertension; Ipc-PH, isolated post-capillary pulmonary hypertension; Cpc-PH, combined pre- and post-capillary pulmonary hypertension.
(TIF)

S1 File Analysis data. (XLSX)
