# Supplementary material for: Combined pre- and post-capillary pulmonary hypertension: The clinical implications for patients with heart failure
Source: PLoS One. 2021 Mar 2;16(3):e0247987. doi: 10.1371/journal.pone.0247987 (PMC7924774; doi:10.1371/journal.pone.0247987)
Supplement: S1 Table — (DOCX) [file pone.0247987.s001.docx]

**S1 Table. Endpoints.**

|  | Total  (n = 701) | Ipc-PH  (n = 268) | Cpc-PH  (n = 54) | Borderline-PH  (n = 112) | Non-PH  (n = 267) | *P* value |
| --- | --- | --- | --- | --- | --- | --- |
| Primary endpoint, n (%) | 166 (23.7%) | 68 (25.4%) | 26 (48.1%) | 25 (22.3%) | 47 (17.6%) | <0.001 |
| Causes of primary endpoint |  |  |  |  |  | <0.001 |
| Cardiac death, n (%) | 24 (3.4%) | 8 (3.0%) | 6 (11.1%) | 3 (2.7%) | 7 (2.6%) |  |
| Re-admission due to heart failure, n (%) | 139 (19.8%) | 58 (21.6%) | 19 (35.2%) | 22 (19.6%) | 40 (15.0%) |  |
| LVAD implantation, n (%) | 3 (0.4%) | 2 (0.7%) | 1 (1.9%) | 0 | 0 |  |

Ipc-PH, isolated post-capillary pulmonary hypertension; Cpc-PH, combined pre- and post-capillary pulmonary hypertension; PH, pulmonary hypertension; LVAD, left ventricular assist device.
