# Supplementary material for: Combined pre- and post-capillary pulmonary hypertension: The clinical implications for patients with heart failure
Source: PLoS One. 2021 Mar 2;16(3):e0247987. doi: 10.1371/journal.pone.0247987 (PMC7924774; doi:10.1371/journal.pone.0247987)
Supplement: S2 Table — (DOCX) [file pone.0247987.s002.docx]

**S2 Table. Multivariate Cox regression analysis to predict primary endpoint using conventional PH criteria and PVR.**

| Variables | HR | 95% CI | *P* value |
| --- | --- | --- | --- |
| Classification of PH |  |  |  |
| Non-PH (vs. Ipc-PH) | 0.66 | 0.46 - 0.94 | 0.02 |
| Borderline-PH (vs. Ipc-PH) | 0.68 | 0.30 - 1.52 | 0.34 |
| Cpc-PH (vs. Ipc-PH) | 1.78 | 1.09 - 2.91 | 0.02 |
| Age (10 year increase) | 1.23 | 1.06 - 1.43 | 0.007 |
| Male sex (vs. female) | 0.97 | 0.68 - 1.37 | 0.84 |
| Overweight (BMI ≥25 kg/m^2^) | 0.98 | 0.70 - 1.38 | 0.92 |
| Systolic blood pressure at admission (10 mmHg increase) | 0.99 | 0.93 - 1.05 | 0.76 |
| Ischemic heart disease | 1.74 | 1.15 - 2.65 | 0.009 |
| Anemia | 1.33 | 0.94 - 1.87 | 0.11 |
| Hyperuricemia | 1.09 | 0.77 - 1.56 | 0.62 |
| Impaired renal function (eGFR <60 ml/min/1.73 m^2^) | 1.08 | 0.77 - 1.51 | 0.66 |
| Atrial fibrillation or flutter | 1.09 | 0.78 - 1.53 | 0.61 |
| Reduced LVEF (vs. preserved LVEF) | 1.23 | 0.85 - 1.77 | 0.27 |
| Loop diuretics use | 1.04 | 0.65 - 1.65 | 0.88 |

PH, pulmonary hypertension; PVR, pulmonary vascular resistance; Ipc-PH, isolated post-capillary pulmonary hypertension; Cpc-PH, combined pre- and post-capillary pulmonary hypertension; BMI, body mass index; eGFR, estimated glomerular filtration rate; LVEF, left ventricular ejection fraction.
