# Supplementary material for: Combined pre- and post-capillary pulmonary hypertension: The clinical implications for patients with heart failure
Source: PLoS One. 2021 Mar 2;16(3):e0247987. doi: 10.1371/journal.pone.0247987 (PMC7924774; doi:10.1371/journal.pone.0247987)
Supplement: S3 Table — (DOCX) [file pone.0247987.s003.docx]

**S3 Table. Multivariate Cox regression analysis to predict primary endpoint using conventional PH criteria and DPG.**

| Variables | HR | 95% CI | *P* value |
| --- | --- | --- | --- |
| Classification of PH |  |  |  |
| Non-PH (vs. Ipc-PH) | 0.60 | 0.43 - 0.84 | 0.003 |
| Borderline-PH (vs. Ipc-PH) | 0.61 | 0.27 - 1.35 | 0.22 |
| Cpc-PH (vs. Ipc-PH) | 1.84 | 0.92 - 3.68 | 0.08 |
| Age (10 year increase) | 1.22 | 1.05 - 1.42 | 0.008 |
| Male sex (vs. female) | 0.93 | 0.66 - 1.33 | 0.70 |
| Overweight (BMI ≥25 kg/m^2^) | 0.96 | 0.68 - 1.35 | 0.80 |
| Systolic blood pressure at admission (10 mmHg increase) | 0.99 | 0.93 - 1.05 | 0.71 |
| Ischemic heart disease | 1.83 | 1.21 - 2.77 | 0.004 |
| Anemia | 1.33 | 0.94 - 1.87 | 0.11 |
| Hyperuricemia | 1.09 | 0.77 - 1.55 | 0.62 |
| Impaired renal function (eGFR <60 ml/min/1.73 m^2^) | 1.10 | 0.78 - 1.53 | 0.59 |
| Atrial fibrillation or flutter | 1.07 | 0.77 - 1.50 | 0.68 |
| Reduced LVEF (vs. preserved LVEF) | 1.22 | 0.84 - 1.76 | 0.29 |
| Loop diuretics use | 1.04 | 0.65 - 1.65 | 0.87 |

PH, pulmonary hypertension; DPG, diastolic pressure gradient; Ipc-PH, isolated post-capillary pulmonary hypertension; Cpc-PH, combined pre- and post-capillary pulmonary hypertension; BMI, body mass index; eGFR, estimated glomerular filtration rate; LVEF, left ventricular ejection fraction.
