# Supplementary material for: Combined pre- and post-capillary pulmonary hypertension: The clinical implications for patients with heart failure
Source: PLoS One. 2021 Mar 2;16(3):e0247987. doi: 10.1371/journal.pone.0247987 (PMC7924774; doi:10.1371/journal.pone.0247987)
Supplement: S4 Table — (DOCX) [file pone.0247987.s004.docx]

**S4 Table. Multivariate Cox regression analysis to predict primary endpoint using the new PH criteria and DPG.**

| Variables | HR | 95% CI | *P* value |
| --- | --- | --- | --- |
| Classification of PH |  |  |  |
| Non-PH (vs. Ipc-PH) | 0.59 | 0.41 - 0.85 | 0.005 |
| Borderline-PH (vs. Ipc-PH) | 0.63 | 0.40 - 0.99 | 0.047 |
| Cpc-PH (vs. Ipc-PH) | 1.88 | 0.95 - 3.75 | 0.07 |
| Age (10 year increase) | 1.21 | 1.05 - 1.41 | 0.01 |
| Male sex (vs. female) | 0.95 | 0.67 - 1.36 | 0.79 |
| Overweight (BMI ≥25 kg/m^2^) | 0.96 | 0.68 - 1.35 | 0.81 |
| Systolic blood pressure at admission (10 mmHg increase) | 0.99 | 0.93 - 1.05 | 0.76 |
| Ischemic heart disease | 1.89 | 1.26 - 2.85 | 0.002 |
| Anemia | 1.35 | 0.96 - 1.90 | 0.09 |
| Hyperuricemia | 1.08 | 0.76 - 1.53 | 0.68 |
| Impaired renal function (eGFR <60 ml/min/1.73 m^2^) | 1.08 | 0.77 - 1.52 | 0.65 |
| Atrial fibrillation or flutter | 1.03 | 0.74 - 1.45 | 0.84 |
| Reduced LVEF (vs. preserved LVEF) | 1.19 | 0.83 - 1.72 | 0.35 |
| Loop diuretics use | 1.02 | 0.64 - 1.62 | 0.94 |

PH, pulmonary hypertension; DPG, diastolic pressure gradient; Ipc-PH, isolated post-capillary pulmonary hypertension; Cpc-PH, combined pre- and post-capillary pulmonary hypertension; BMI, body mass index; eGFR, estimated glomerular filtration rate; LVEF, left ventricular ejection fraction.
