# Supplementary material for: Combined pre- and post-capillary pulmonary hypertension: The clinical implications for patients with heart failure
Source: PLoS One. 2021 Mar 2;16(3):e0247987. doi: 10.1371/journal.pone.0247987 (PMC7924774; doi:10.1371/journal.pone.0247987)
Supplement: S5 Table — (DOCX) [file pone.0247987.s005.docx]

**S5 Table. Multivariate Cox regression analysis to predict primary endpoint using the current PH-LHD definition by the 2015 ESC/ERS Guidelines for the diagnosis and treatment of pulmonary hypertension.**

| Variables | HR | 95% CI | *P* value |
| --- | --- | --- | --- |
| Classification of PH |  |  |  |
| Non-PH (vs. Ipc-PH) | 0.66 | 0.46 - 0.95 | 0.02 |
| Borderline-PH (vs. Ipc-PH) | 0.68 | 0.30 – 1.52 | 0.34 |
| Cpc-PH (vs. Ipc-PH) | 1.76 | 1.08 – 2.85 | 0.02 |
| Age (10 year increase) | 1.23 | 1.06 - 1.43 | 0.007 |
| Male sex (vs. female) | 0.96 | 0.68 - 1.37 | 0.83 |
| Overweight (BMI ≥25 kg/m^2^) | 0.97 | 0.69 - 1.37 | 0.87 |
| Systolic blood pressure at admission (10 mmHg increase) | 0.99 | 0.93 - 1.06 | 0.79 |
| Ischemic heart disease | 1.76 | 1.16 - 2.67 | 0.008 |
| Anemia | 1.32 | 0.94 - 1.86 | 0.12 |
| Hyperuricemia | 1.10 | 0.77 - 1.56 | 0.60 |
| Impaired renal function (eGFR <60 ml/min/1.73 m^2^) | 1.08 | 0.77 - 1.51 | 0.66 |
| Atrial fibrillation or flutter | 1.09 | 0.78 - 1.53 | 0.61 |
| Reduced LVEF (vs. preserved LVEF) | 1.22 | 0.85 - 1.76 | 0.29 |
| Loop diuretics use | 1.03 | 0.65 - 1.65 | 0.89 |

PH-LHD, pulmonary hypertension due to left heart disease; PH, pulmonary hypertension; DPG, diastolic pressure gradient; Ipc-PH, isolated post-capillary pulmonary hypertension; Cpc-PH, combined pre- and post-capillary pulmonary hypertension; BMI, body mass index; eGFR, estimated glomerular filtration rate; LVEF, left ventricular ejection fraction.
