# Supplementary figures and images for: Combined pre- and post-capillary pulmonary hypertension: The clinical implications for patients with heart failure
Source: PLoS One. 2021 Mar 2;16(3):e0247987. doi: 10.1371/journal.pone.0247987 (PMC7924774; doi:10.1371/journal.pone.0247987)

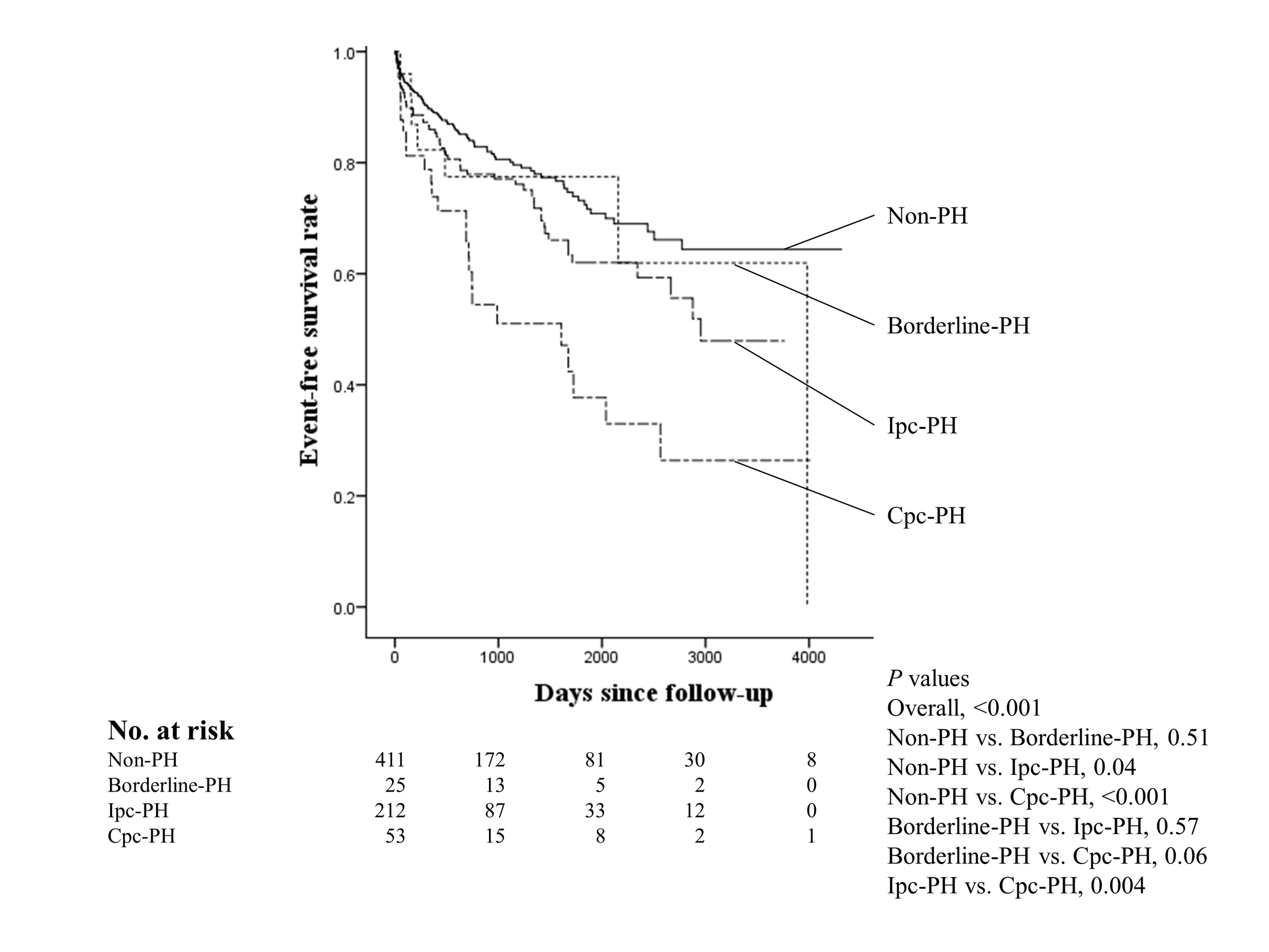

Supplement: S1 Fig — Comparison of the survival curves was performed using the log-rank test. PH, pulmonary hypertension; PVR, pulmonary vascular resistance; Ipc-PH, isolated post-capillary pulmonary hypertension; Cpc-PH, combined pre- and post-capillary pulmonary hypertension. (TIF) [file pone.0247987.s006.tif]

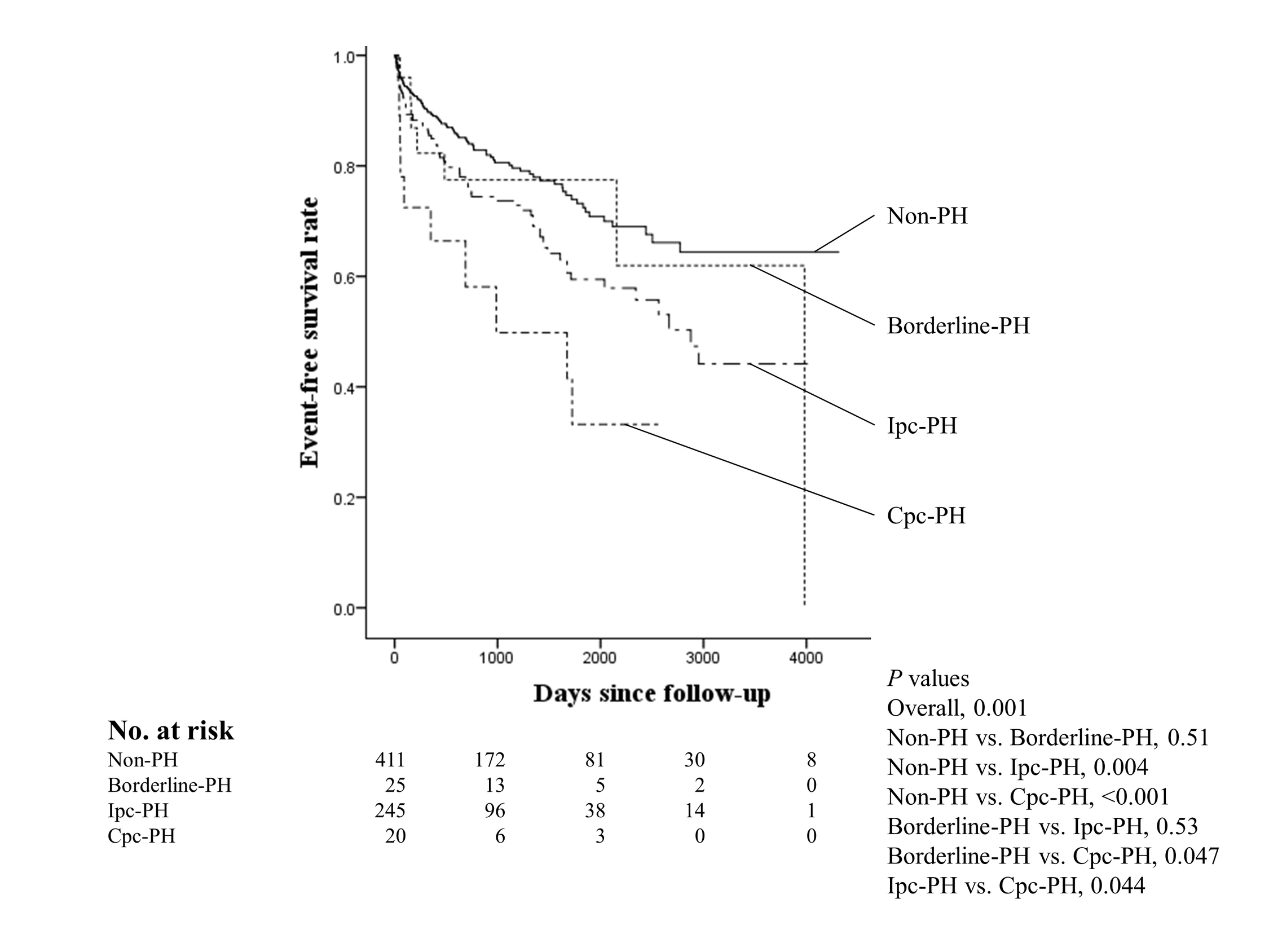

Supplement: S2 Fig — Comparison of the survival curves was performed using the log-rank test. PH, pulmonary hypertension; DPG, diastolic pressure gradient; Ipc-PH, isolated post-capillary pulmonary hypertension; Cpc-PH, combined pre- and post-capillary pulmonary hypertension. (TIF) [file pone.0247987.s007.tif]

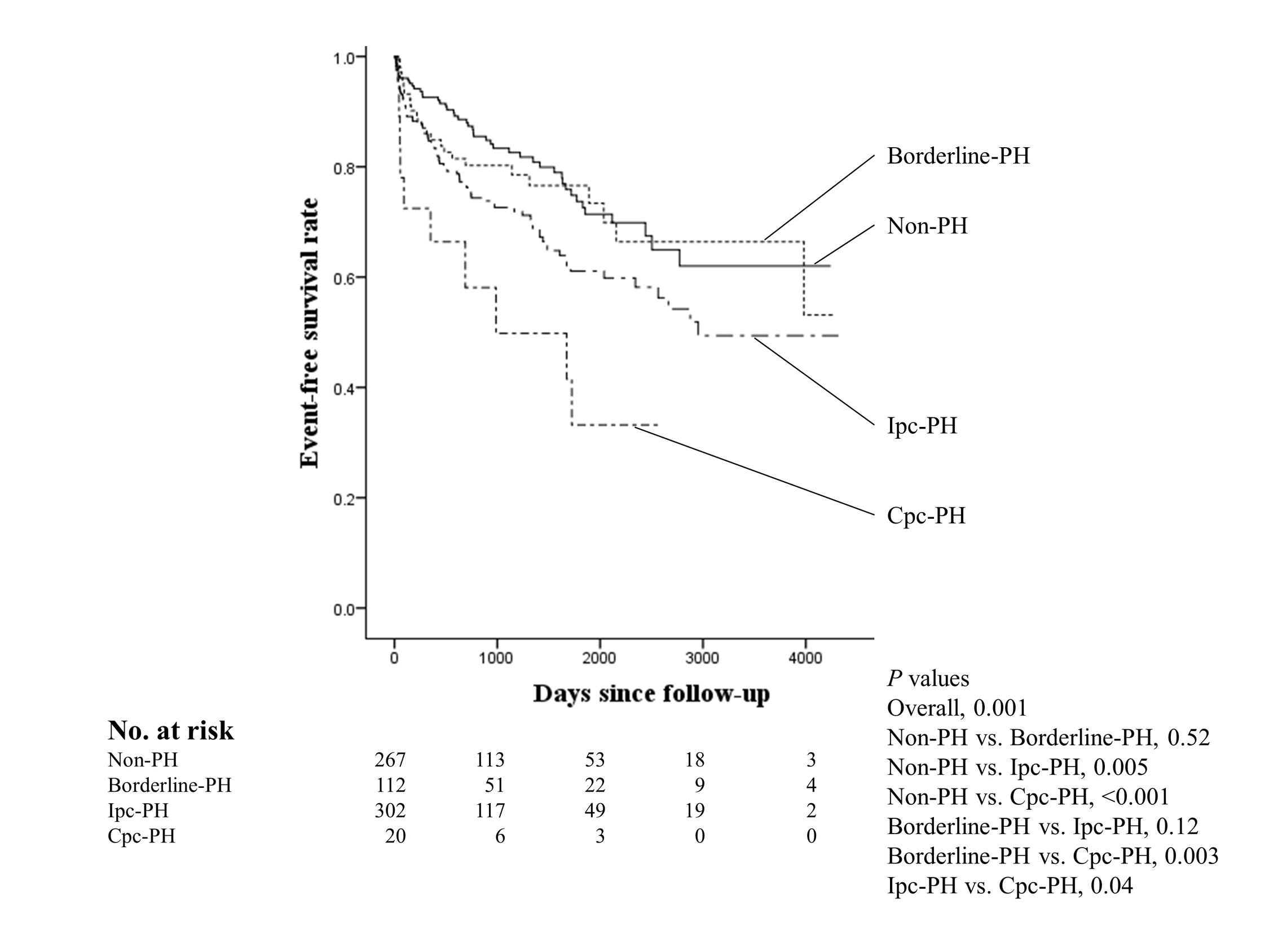

Supplement: S3 Fig — Comparison of the survival curves was performed using the log-rank test. PH, pulmonary hypertension; DPG, diastolic pressure gradient; Ipc-PH, isolated post-capillary pulmonary hypertension; Cpc-PH, combined pre- and post-capillary pulmonary hypertension. (TIF) [file pone.0247987.s008.tif]

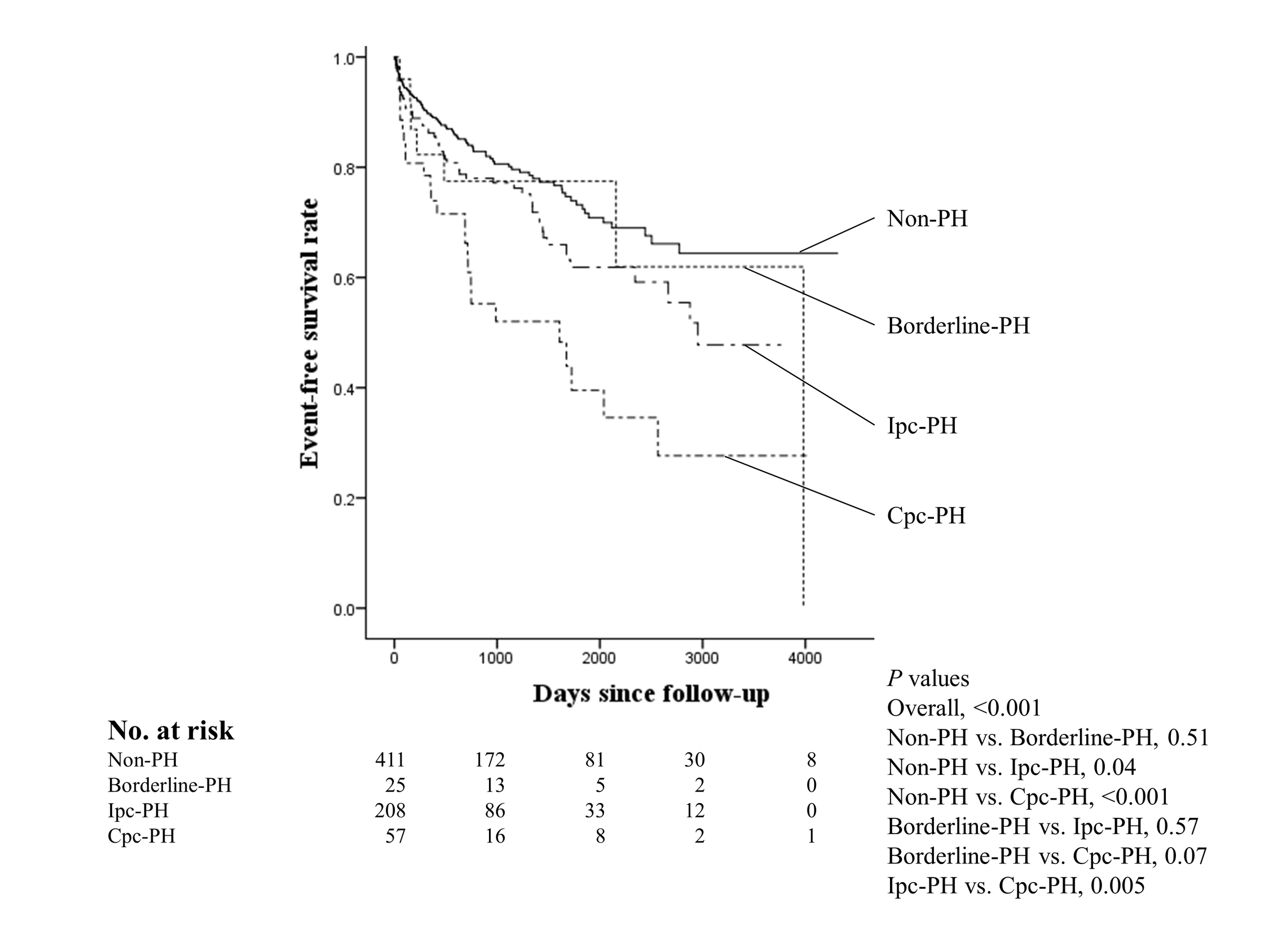

Supplement: S4 Fig — Comparison of the survival curves was performed using the log-rank test. PH-LHD, pulmonary hypertension due to left heart disease; PH, pulmonary hypertension; Ipc-PH, isolated post-capillary pulmonary hypertension; Cpc-PH, combined pre- and post-capillary pulmonary hypertension. (TIF) [file pone.0247987.s009.tif]
